# W-SITTING IN CHILDHOOD: A SYSTEMATIC REVIEW

**DOI:** 10.1590/1413-785220243206e279277

**Published:** 2025-01-10

**Authors:** David Gonçalves Nordon, Caroline de Gouveia Buff Passone, Clovis Artur Almeida da Silva, Patrícia Moreno Grangeiro

**Affiliations:** 1. Pontifícia Universidade Católica de São Paulo, Departamento de Cirurgia, Disciplina de Ortopedia, Sorocaba, SP, Brazil.; 2. Universidade de São Paulo, Faculdade de Medicina, Hospital das Clinicas HC-FMUSP, Departamento de Pediatria, São Paulo, SP, Brazil.; 3. Universidade de São Paulo, Faculdade de Medicina, Hospital das Clinicas HC-FMUSP, Instituto de Ortopedia e Traumatologia, Departamento de Ortopedia, São Paulo, SP, Brazil.

**Keywords:** Child Development Deviation, Sitting Position, Systematic Review, W-sitting, Desvios do Desenvolvimento Infantil, Posição de Sentar, Revisão Sistemática, Sentar em W

## Abstract

Objective: Despite the lack of science-based evidence, many specialists and non-specialists consider W-sitting detrimental to children. This systematic review aims to find evidence on W-sitting. Methods: This review was registered on PROSPERO under the number CRD42022313341. During January 2023, the term “W-sitting” and its variations were searched on the following databases: PubMed, Medline, Embase, PEDro, and Cochrane. Duplicate articles and those that addressed themes other than W-sitting were removed. Results: This review found 3641 articles, removed 614 duplicates, and excluded 3021 for focusing on subjects other than W-sitting. It included seven studies for analysis, one of which was a narrative review and two were methodologically inadequate cross-sectional to evaluate the causal effect in W-sitting. Another article evaluated muscular activation in adults according to sitting position. The last article found no causal relation between W-sitting and developmental dysplasia of the hip. Conclusion: This review found no scientific evidence advise against W-sitting in children and no association with hip dysplasia. Moreover, muscular activation remains the same, regardless of the position chosen for sitting. *Level of evidence III, review article.*

## RESUMO

Objetivos: Apesar da falta de evidências científicas, muitos especialistas e não-especialistas consideram danoso para a criança sentar-se em W. Esta revisão sistemática tem o objetivo de encontrar evidências científicas sobre o tema. Métodos: esta revisão está registrada no PROSPERO sob o número CRD42022313341. Durante o mês de janeiro/2023, o termo “W-sitting” e suas variações foram buscadas nas seguintes bases de dados: PubMed, Medline, Embase, PEDro e Cochrane. Artigos duplicados e sem relação com o tema foram removidos. Resultados: 3641 artigos foram encontrados, 614 duplicados foram removidos, e 3021 foram excluídos por não terem relação com o tema. Sete foram incluídos para análise. Um artigo era uma revisão narrativa. Dois artigos eram transversais, metodologicamente inadequados para avaliar os efeitos causais de sentar em W. Outro artigo avaliou a ativação muscular em adultos de acordo com a posição em que se senta. O artigo final concluiu que não há relação causal entre se sentar em W e displasia do desenvolvimento do quadril. Conclusão: não foi encontrada evidência científica para se recomendar evitar que uma criança se sente em W; não há associação com displasia do quadril; a ativação muscular é a mesma, independentemente da posição de sentar. *Nível de evidência III, artigo de revisão.*

## INTRODUCTION

 W-sitting or “television sitting” is a position in which children perform flexion, adduction, and maximal internal rotation at the hip when sitting on the floor. From above, their legs make the shape of a W. Children aged from three to six years usually prefer it as it offers more comfort for those with increased joint mobility and anteversion of the proximal femur (the degree the femoral neck and head point transversally forward), which occurs physiologically at this age. ^1^ ^,^ ^2^ In contrast, sitting in a cross-legged position is more natural for those with decreased femoral anteversion or for whom sitting in a W shape would be painful or impossible. ^1^ ^,^ ^2^

 Despite the lack of science-based evidence against this sitting position, most physical therapists, occupational therapists, teachers, and several other healthcare and education professionals advise against it; a sentiment present across multiple internet sources. ^3^ ^-^ ^6^

This systematic review aims to identify any evidence for or against W-sitting that warrants consideration when advising for or against this sitting position.

## METHODS

This review is registered on PROSPERO under the number CRD42022313341.

**Figure 1.**
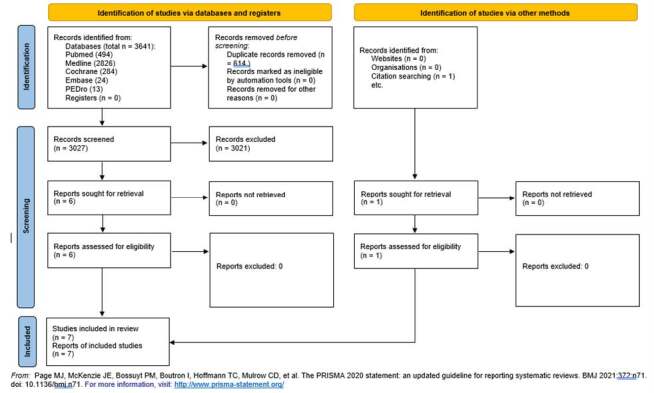
Flow diagram for systematic review.

 During January 2023, two independent researchers (DN and CP) performed a search on the PubMed, Medline, Cochrane Library, Embase, and PEDro databases by using the terms “W-sitting,” “W sitting,” “Television sitting,” and “W-shape sitting.” The results according to those keywords are shown in Table 1 and Figure 1 . A third reviewer (PM) reviewed the articles considered for inclusion. 

**Table 1.** Findings according to keywords.

Keyword/Database	PubMed	Medline	Cochrane	Embase	PEDro
W-sitting	6 (3 obtained)	2 (1 new article)	1 (0 obtained)	9 (4 found, 1 retrieved)	4 (0)
W sitting	487 (5, 1 new article)	2804 (no new articles)	223 (0 obtained)	9 (5 found, 2 retrieved)	4 (0, no new articles)
Television sitting	0	20 (0)	60 (0 obtained)	5 (1 found, 0 retrieved)	5 (1 new, 0 retrieved)
W-shape sitting	1 (no new articles)	0	0	1 (1 found, 0 retrieved)	0

A search on Google Scholar was also performed. However, most of its articles were opinion texts and content from blogs or websites — including no clinical studies other than the ones that had already been found on other platforms. Therefore, this search was ignored in the systematic review.

The following inclusion criteria were considered: the article must concern W-sitting. Clinical studies were preferred. Still, reviews, case reports, and even specialists’ opinions were included. No language or date limitations were applied.

Articles were only excluded from the analysis if they ignored W-sitting in any way. For that, the researcher considered both the title and the abstract; in case of doubts, the article was obtained and thoroughly read. The reviewers showed no disagreements regarding article inclusion.

 All relevant information was obtained and included on a Microsoft Excel ^TM^ spreadsheet. 

 After data synthesis and studies categorization as described above, the final report was prepared following the Preferred Reporting Items for Systematic Reviews and Meta-Analyses (PRISMA) guidelines. ^7^ Risk of bias was described according to the ROBINS I tool for non-randomized studies. ^8^

 When possible, the articles in this review were also evaluated using the Bradford-Hill criteria ^9^ to determine causality of a medical condition: strength of association (the observed association between two variables, such as relative risk), consistency (repeating the same test obtains similar results), specificity (cause is specific to the effect, removing the cause reverses or eliminates the effect), temporality (effect comes after its cause), biological gradient (optional, but the more the patient is exposed to a specific variable, the more effect it causes), plausibility (whether the cause and the effect are biologically plausible), coherence (whether their association is coherent with current medical knowledge), experiment (causation proved by earlier experiments), and analogy (if the causative association is possible by analogy to other similar associations). Healthcare providers should use these criteria when considering whether W-sitting may cause any adverse effects. 

## RESULTS

 The first search strategy was to use the terms “W-sitting” and “W sitting” as keywords. This review selected and retrieved six articles ^10^ ^-^ ^15^ . A thorough reading and reference review found one more article. Altinel et al. ^16^ used the term “Television sitting,” which was also used as a new search strategy, but it obtained no other articles. Lee et al. ^11^ used the term “W-shape.” Thus, we performed a new search using “W-shape sitting” as a new keyword but it retrieved no other articles. 

The number of findings using “W-sitting” and “W sitting” as keywords considerably differ for the search engine considers “W” and “Sitting” as two different words in the latter, thus retrieving articles that use “W’ mostly as an abbreviation for”Women.” Thus, using “W Sitting” as a new keyword obtained no new articles, and further research may be performed by using solely the term “W-sitting.”

Table 2 shows the seven articles included for analysis: 

**Table 2.** Characteristics and most relevant information of each article obtained for analysis.

Main author and year of publication	Methods	Results	Considerations
Altinel, 2007. ^16^	Study design: Cross-sectional, observational study. Variables: Evaluation of gait, in-toeing, hip range of movement (ROM) and sitting habits. Population: 1134 children aged from three to six years.	5.9% of children showed in-toeing, and 75% were bilateral. In-toeing was 2.4 times more common in girls, and 75% were related to femoral anteversion. It is associated with increased internal rotation and decreased external rotation of the hips. 37% of the children sat in a cross-legged position, and 63% in W-shape. W-sitting was associated with in-toeing (p=0.001)	As in any cross-sectional study, it is possible to associate W-sitting with in-toeing but not to infer causality.
Chen, 2010. ^10^	Study design: Cross-sectional, observational study. Variables: Evaluation of feet arches, joint laxity, and W-sitting. Population: 1538 children.	29.7% of children showed joint laxity and 17.3% W-sat. 46.7% of the children with bilateral flat feet presented with joint laxity and 41.2% of those with bilateral flatfeet W-sat.	Despite no evident causality, the authors consider W-sitting as a cause for flatfeet.
Lee, 2017. ^11^	Study design: Cross-sectional, observational study. Variables: Muscular activation (external oblique, rectus abdominis, latissimus dorsi, erector spinae) in four sitting positions (cross-legged, side, long, W-shaped) Population: 8 adults.	No significant difference in muscle activation in the evaluated sitting positions: cross-legged, side, long, W-shaped.	Empirical evidence that W-sitting leads to no abnormal muscle activation and, therefore, development (by extrapolation). The authors concluded that more studies are necessary.
Goldstein, 2019. ^12^	Study design: Retrospective cohort. Variables: developmental dysplasia of the hip; W-sitting status and duration; hip angles. Population: 27 children (11 W-sitters and 16 non-W-sitters).	No correlation between W-sitting and developmental dysplasia of the hip.	Meeting abstract; preliminary findings for the article below (Rethlefsen, 2020).
Rethlefsen, 2020. ^13^	Study design: Retrospective cohort. Variables: developmental dysplasia of the hip; W-sitting status and duration; hip angles. Population: 104 children (18 with and 86 without hip dysplasia); 48 W-sat.	No correlation between W-sitting and developmental dysplasia of the hip.	Compatible with clinical findings in the pediatric orthopedics.
Honig, 2021. ^14^	Study design: Narrative review. No specification of literature research methodology.	Considers that W-sitting should not be prohibited.	Most arguments were based on literature on normal child development and on Rethlefsen 2020.
Lamari, 2022. ^15^	Study design: Retrospective cohort. Variables: psychosocial and characteristics of patients with joint hypermobility. Population: 482 children and adults with hypermobility.	Around 55% of patients with joint hypermobility are or were able to W-sit.	Authors associated W-sitting with no complications.

 The level of evidence in the included articles — three cross-sectional observational studies, three retrospective cohort studies, and one narrative review — is considerably low. Only one (considering Goldstein ^12^ and Rethlefsen ^13^ as the same study) had an appropriate methodology to investigate causation between W-sitting and orthopedic conditions or complications. The lack of good data became more evident by evaluating the articles by the Bradford-Hill criteria ( Table 3 ). ^9^ This review ignored the analysis of two articles (Honig et al. ^14^ and Goldstein et al.) ^12^ by the Bradford-Hill criteria. Honig et al. ^14^ was unsuitable for them and Goldstein et al. ^12^ was only an abstract of the complete study in Rethlefsen et al. ^13^

Most studies in this review inadequately proved causality between W-sitting and complications according to the Bradford Hill criteria.

**Figure 2.**
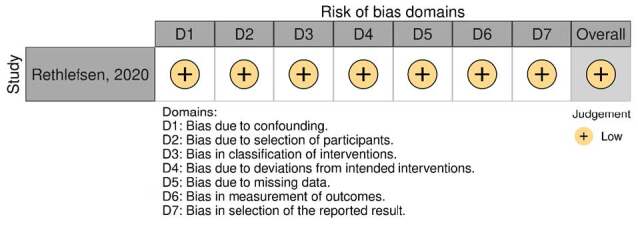
ROBINS I tool for non-randomized studies for W-sitting.

 Finally, this review applied he ROBINS I tool ^8^ for assessing risk of bias in non-randomized studies to Rethlefsen ^13^ since it was the only suitable article for such an analysis (a retrospective cohort designed to specifically assess the association between W-sitting and developmental dysplasia of the hip [DDH]). It showed a low risk of bias. ^13^ ( Figure 2 ). 

**Table 3.** Bradford Hill Criteria applied on the articles included in this systematic review.

Criteria	Altinel, 2007 ^16^	Chen, 2010 ^10^	Lee, 2017 ^11^	Rethlefsen, 2020 ^13^	Lamari, 2022 ^15^
Strength	Cross-sectional studies can calculate prevalence ratios, rather than risk ratios. This type of study cannot prove causality.			Cohort studies can calculate relative risk. Strength is higher.	
Consistency	Although all these studies are reproducible, none have been reproduced in other populations yet.				
Specificity	The methodologies of the studies are insufficient for a specific association between cause and effect (factors of confusion remained uncontrolled).		The design of the chosen studies sufficiently showed specificity.		This study was not designed to evaluate W-sitting.
Temporality	Impossible to assert that cause precedes effect in time due to the observational study design.			Adequate methodology in study design (retrospective cohort).	
Biological gradient	The design of the chosen studies are inadequate for biological gradient evaluation.			Adequate methodology (current and past W-sitters).	The design of the chosen studies is inadequate for biological gradient evaluation.
Plausibility	Findings are implausible with the current medical knowledge.		Findings are plausible with current medical knowledge.		
Coherence	Association is incoherent with the current medical knowledge.		Association is coherent with current medical knowledge.		
Experiment	The methodologies of the studies prohibit causality tests.			Can test for causality, but a prospective study would be preferable	This study was not designed to evaluate causality in W-sitting.
Analogy	Analogies are incompatible with current medical knowledge.		Analogies are compatible with current medical knowledge.		

## DISCUSSION

The discussion of W-sitting and its detrimental effects on children is old and persistent. Finding the origin of such assumptions offers a difficult task. We suspect anecdotes in which the habit of W-sitting was initially associated with muscular abnormalities in cerebral palsy patients formed the basis of a belief in the harmful effects of W-sitting. This belief was then likely extended to the neurotypical population without adequate scientific evidence. Most arguments against W-sitting lack empirical evidence or use evidence improperly, as this systematic review has shown.

 Out of the six clinical studies included in this systematic review, two associated W-sitting with other orthopedic “complications” (in-toeing [walking with the feet pointed medially] in Altinel ^16^ and flatfeet in Chen ^10^ ); two repudiated such an association (Goldstein ^12^ and Rethlefsen ^13^ ); two ignored this relationship (Lamari ^15^ and Lee ^11^ ), although the latter (Lee) may be considered as arguing against such an association. 

 Altinel et al. ^16^ considered W-sitting as a cause for in-toeing. However, not only was the study design inadequate to infer such causality, but it also implausibly considered our current knowledge of the physiological development of children’s limbs. All children are born with increased femoral anteversion, which slowly decreases during life, until around 10 years of age. ^1^ ^,^ ^2^ ^,^ ^17^ During normal gait development, the tension of iliofemoral ligaments and muscular recruitment shape the proximal femur into a final anteversion of around 15°. Children with delayed or impaired gait have a persistent femoral anteversion, as in several studies in cerebral palsy patients. ^18^ ^,^ ^19^ In typical development, as children grow and femoral anteversion decreases, they tend to stop W-sitting. 

 Children with increased femoral anteversion enjoy greater comfort W-siting as it increases internal rotation of the hip, whereas a cross-legged position offers more comfort for those with decreased femoral anteversion. ^1^ ^,^ ^2^ Moreover, ligament laxity during infancy may also contribute to an increased range of motion at the hip, raising internal rotation and W-sitting, as in Lamari et al. ^15^ Ligament laxity may be restricted to a specific joint (or more than one) or may affect every or most tissues, thus causing a hypermobility syndrome. 

 Chen et al. ^10^ intended to correlate joint laxity, flatfeet, and W-sitting. Again, their cross-sectional study was unable to prove causality. From a biological and mechanical point of view, it is more plausible to consider that a hypermobile hip joint would enable children to more easily W-sit and increase the flexibility of their foot arches. The medical literature has described that the medial longitudinal arch of the feet starts its development by the age of two years, ending at age from seven to 10 years. ^20^ Up to two years of age, almost all children show flatfeet; and from then on, its prevalence gradually decreases, as does that of joint laxity. ^21^ ^,^ ^22^ Chen et al. ^10^ described the decrease in flatfeet prevalence to happen from ages three to six years by observing children of different ages, rather than by a longitudinal observation. Finally, the principle of temporality discredits Chen’s theory; flatfeet occur universally, even before children begin to W-sit and progressively decrease by the time they W-sit. So, following the authors’ logic, one might consider that W-sitting “cures” flatfeet. 

 Similarly, as in Altinel et al., ^16^ biological and mechanical plausibility should be considered: is it more likely that W-sitting causes flatfeet or is it more likely that a more intense joint laxity both enable children to W-sit and predisposes them to flat feet? ^16^ By reviewing the data from their own study, the authors might be able to determine the answer. However, they ignored such analysis. 

 Lee et al. ^11^ evaluated muscular activation (i.e., the muscles required during a certain action) in different sitting positions. This is an important analysis since several healthcare providers state (despite lack of evidence) that W-sitting will compromise the development of trunk muscles and children’s ability to turn and execute functions with crossed arms (as, for instance, picking up a toy on their left side with their right hand). ^14^ This study was performed on adults due to the challenge of performing electromyographic analysis in children. Therefore, their findings should not be applied to children without further considerations. Nevertheless, there should exist no reason to think that muscular activation in children would be so different as to render this study irrelevant to children (considering analogy, coherence, and plausibility). 

 The last study this systematic review included refers to Rethlefsen et al. ^13^ Goldstein et al. ^12^ reported the preliminary findings from Rethlefsen et al. ^13^ The authors performed a retrospective cohort study with 104 children who had received a pelvic X-ray. The patients’ parents were asked whether their children used to W-sit and if they still W-sat, for how long they sat in such a position, and when they stopped W-sitting if they stopped. In total, 48 children W-sat. Those authors then analyzed the X-rays of the entire study population were to assessment their center edge angle and acetabular index (measures required for diagnosing DDH in children via X-ray), showing that 18 children had DDH. There was no correlation between W-sitting and DDH. This review analyzed this article by the ROBINS I tool and found its low risk of bias. 

This systematic review has some limitations. Despite its comprehensive literature review, the indexed journals published only a few articles. This might lead to a publication bias by disregarding articles published in non-indexed journals. Nevertheless, indexed journals are in effect the highest level of evidence, and the inclusion of non-indexed articles might lead to yet another bias – and thus we decided to ignore them.

After conducting this systematic review and considering the lack of scientific evidence , biological and mechanical plausibility, coherence, and analogy in the studied articles, the authors of this review think that W-sitting fails to harm children’s hips or development, and should thus face no restriction. W-sitting occurs commonly in children and rarely in adulthood due to the physiological development of femoral anteversion, which naturally regresses and makes it mechanically impossible to W-sit regardless of postural correction or physical therapy. However, new longitudinal studies should be conducted with adequate methodologies (prospective cohort or a clinical trial) and a large multicentric and multinational population. Furthermore, these future studies should include uniform musculoskeletal criteria for joint hypermobility, flatfeet, DDH, and muscular activation.

## CONCLUSION

W-sitting shows no relation with evidence-based developmental dysplasia of the hip. No scientific evidence suggests any other orthopedic deformity. W-sitting results in no evidence-based different trunk muscle activation. More studies must be performed since the current studies show a predominantly low level of evidence and methodological inadequacies to prove harm in W-sitting. Therefore, prohibiting W-sitting in children lacks evidence.
